# Optimization of rotor-side controller parameters in doubly fed induction generators based on an improved NSGA-II

**DOI:** 10.1371/journal.pone.0326077

**Published:** 2025-06-23

**Authors:** Yanling Lv, Xiang Zhao, Zexin Mou

**Affiliations:** 1 School of Electrical and Electronic Engineering, Harbin University of Science and Technology, Harbin, Heilongjiang Province, China; 2 State Grid Heilongjiang Electric Power Co., Ltd., Electric power Research Institute, Harbin, Heilongjiang Province, China; Aalto University, FINLAND

## Abstract

Herein, an advanced control strategy to enhance the operational stability of wind turbine generators during grid-voltage surges is presented. In particular, a multiobjective optimization framework based on an improved nondominated sorting genetic algorithm II (NSGA-II) is proposed by establishing a dynamic model of the rotor-side converter and investigating the operational dynamics of proportional–integral–derivative controllers under voltage transients. Comparative simulations using the traditional NSGA-II, a multiobjective particle swarm optimization algorithm, and a multiobjective gray wolf optimization algorithm are conducted to validate the proposed algorithm. The improved NSGA-II exhibits superior robustness in suppressing equipment wear and minimizing harmonic distortions under transient conditions. These advancements highlight the potential of the proposed framework for enhancing grid resilience and operational efficiency in wind power systems.

## Introduction

The development of modern power systems has resulted from the evolution of energy sources and artificial intelligence. Moreover, innovations in digitalization and intelligent technologies have globally driven the transition toward safer, more efficient, greener, and low-carbon systems. Wind power has garnered considerable research attention as a clean and renewable energy source [1]. Doubly fed induction generators (DFIGs), with high efficiency, flexible operation, and grid-friendly characteristics, have become one of the mainstream technologies in the wind power sector [2]. DFIGs can be used in large-scale wind farms owing to their unique rotor-side frequency control technology and excellent grid adaptability [3]. The grid integration of wind power has necessitated the use of control strategies for managing the nonlinear, uncertain, and dynamic characteristics of wind power systems. The ability of wind power systems to maintain stable operation during grid faults and voltage fluctuations directly affects the overall security of power grids. Hence, high-voltage ride-through (HVRT), an essential indicator used for evaluating the stability of wind power systems, has garnered substantial research attention. The HVRT capability enables wind turbines to maintain a stable connection with the grid during brief voltage spikes without generating excessive transient currents, ensuring their stable operation and equipment safety. During high-voltage faults in the power grid, the rotor-side converter of DFIGs experiences substantial current fluctuations due to a sudden increase in voltage. These fluctuations can reduce the stability of power systems and severely damage equipment. Excessive currents may cause converter overheating and insulation damage, potentially causing grid disconnection. Therefore, effectively controlling current transients during HVRT events is one of the core issues that needs consideration in studies on current wind power systems.

The rotor-side controller parameters of DFIGs considerably influence their operational performance and stability. During grid faults and other disturbances, the rotor-side controller parameters directly affect the low-voltage ride-through (LVRT) capability of DFIGs and overall system stability [4]. During HVRT events, the rotor-side controller must rapidly limit excessive rotor-side currents to avoid any damage to power electronic devices. Improperly set controller parameters can cause system oscillations or failure in realizing normal operation [5]. The selection and optimization of rotor-side controller parameters are crucial for enhancing the operational performance and stability of DFIGs. These parameters can be fine-tuned using advanced optimization algorithms to considerably improve the dynamic performance and disturbance resistance of DFIGs. After controller parameter optimization, DFIGs can adapt to complex operational environments and meet the stringent requirements of modern power grids [6]. The inherent fluctuations and unpredictability in the output of renewable-energy-based power generation systems complicate maintaining grid balance. Intelligent algorithms have played a critical role in overcoming these issues. Fuzzy logic control, fuzzy-neural-network-based control, and deep reinforcement learning have been employed to regulate power output and manage energy storage systems. These methods effectively mitigate the output fluctuations of wind- and solar-energy-based power generation systems. Algorithms that combine adaptive fuzzy control with proportional–integral–derivative (PID) control can dynamically adjust the charge and discharge rates of energy storage systems, reducing the effect of renewable energy fluctuations on power grids. Deep learning and reinforcement learning algorithms have demonstrated considerable potential in wind power forecasting and short-term fluctuation smoothing. However, the foundational theoretical innovations and strategic improvements in these intelligent algorithms remain inadequate. Moreover, most current advancements primarily focus on power optimization and load forecasting, lacking an optimization strategy that specifically targets rotor-side current characteristics and harmonic suppression in wind turbines.

Yessef et al. [7] reported an improved robust control strategy for the rotor-side converter of a wind energy conversion system. The strategy combined direct power control and backstepping control to enhance energy extraction and ensure high system performance and robustness in different operational modes. Yessef et al. [8] also introduced two control strategies, namely field-oriented control and an adaptive backstepping method, to optimize wind energy extraction and DFIG performance under variable wind speeds. Yessef et al. [9] further investigated two robust control strategies, adaptive backstepping and integral sliding mode control, to mitigate high peak currents and stabilize active power during grid disturbances such as voltage sags. Yessef et al. [10] reported an improved direct power control strategy based on backstepping for maintaining normal DFIG operation during grid faults. Yessef et al. [11] explored the combined use of direct power control and a neural super-twisting algorithm to regulate the DFIG power output and thereby minimize its fluctuations. Benbouhenni et al. [12] proposed a dual super-twisting sliding mode control strategy integrated with pulse width modulation to replace conventional direct power control for rotor-side converters in multirotor wind systems. Benbouhenni et al. [13] introduced a hybrid neuro-fuzzy fractional-order control strategy integrated with pulse width modulation to optimize the power output of DFIG-based dual-rotor wind turbines. Li et al. proposed an improved method for the dynamic self-adaptive control of crowbar resistance based on traditional crowbar circuits applicable during LVRT and HVRT events [14]. Zhang et al. combined three measures to enhance the HVRT capability of DFIGs in wind power systems: series connection of stator-side reactance, addition of a discharge circuit on the DC side, and reactive power output control of the grid-side converter [15]. Zhong et al. achieved improved HVRT performance by adaptively adjusting the reference value of the DC-link voltage in grid-side converters [16]. Sun et al. achieved enhanced HVRT performance by considering the effect of dynamic variations in stator flux linkages on the power outer loop and leveraging the concept of passive damping protection [17]. Xu et al. employed a coordinated control strategy for rotor-side and grid-side converters to enhance the HVRT capability of DFIGs [18].

Existing research on DFIG control strategies has made considerable progress in improving system transient performance and fault tolerance using advanced methods such as backstepping and sliding mode control. However, studies have often overlooked systematic parameter optimization for rotor-side controllers, resulting in suboptimal dynamic responses during grid disturbances, including excessive overshoot, prolonged oscillations, and delayed recovery under fault conditions. Furthermore, conventional optimization methods struggle to balance competing objectives such as transient current suppression, dynamic response acceleration, and steady-state stability, particularly in high-dimensional, nonlinear systems. These limitations hinder the operational reliability of wind turbines under complex grid conditions and increase the risk of mechanical wear caused by repeated current surges.

To address the aforementioned challenges, this study proposes a PID controller parameter optimization method based on an improved nondominated sorting genetic algorithm II (NSGA-II). This algorithm integrates a multiobjective optimization strategy with two novel mechanisms: automatic parameter tuning for the adaptive identification of optimal PID gains and a knight elite strategy to retain high-quality solutions during exploration. By simultaneously optimizing the control accuracy, dynamic response speed, and overshoot suppression under fault conditions, the algorithm achieves a precise balance between transient performance and steady-state stability. Comparative simulations demonstrate that the optimized PID controller considerably reduces rotor-side current overshoot during voltage surges, accelerates post-fault recovery, and mitigates mechanical stress on the converter, which are critical factors for achieving real-world durability but are often neglected in theoretical studies. By bridging the gap between algorithmic innovation and practical parameter optimization, this study advances the robustness and adaptability of DFIG systems under grid disturbances, offering a scalable solution for modern wind energy applications.

This remainder of this manuscript is structured as follows:

Section 2: DFIG Model—This section presents the physical and mathematical models of a DFIG, laying the foundation for subsequent development of a control strategy.

Section 3: Rotor-side Converter Control Strategy Based on the Improved NSGA-II—This section introduces the working principle of a rotor-side converter and details the optimization algorithm and improvement strategy, focusing on enhancing system performance using the improved NSGA-II.

Section 4: Simulation Analysis—This section presents a comparative analysis of the improved NSGA-II against the traditional NSGA-II, a multiobjective particle swarm optimization (MOPSO) algorithm, and a multiobjective gray wolf optimization (MOGWO) algorithm, highlighting the effectiveness of the proposed method through simulation experiments.

Section 5: Conclusion—This section summarizes the key findings, highlights the advantages of the proposed method, and discusses potential directions for future research.

## DFIG model

**Fig 1:**
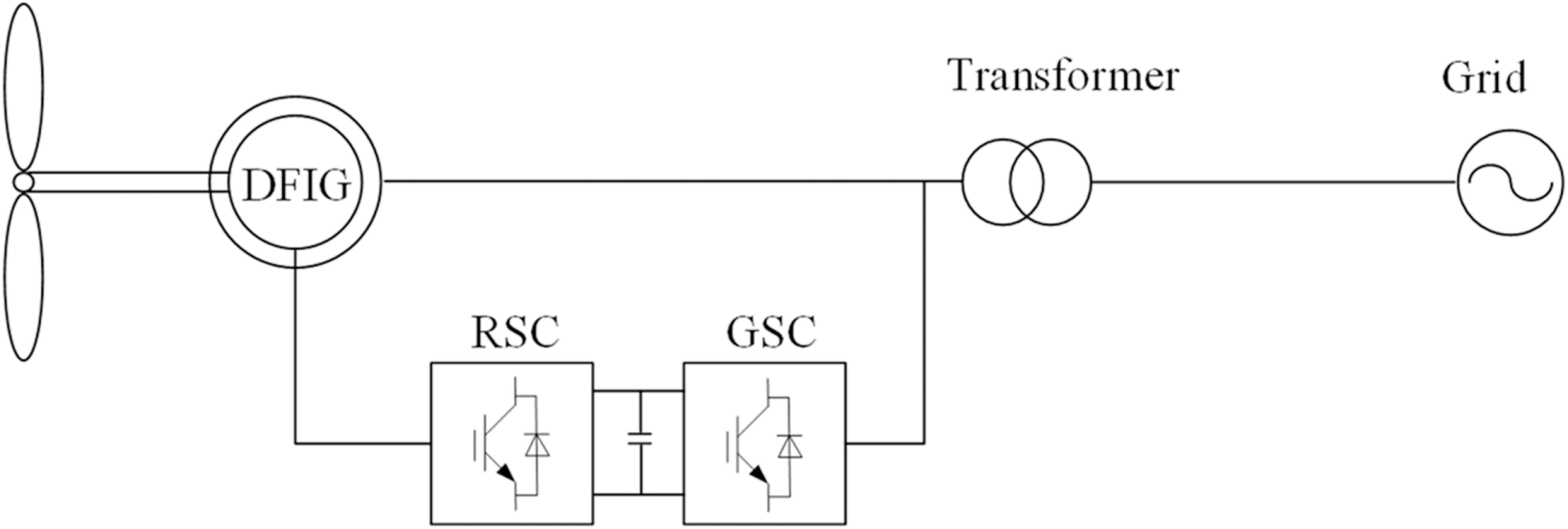
Structure of a DFIG.

An aerodynamic model is used to describe the dynamics of wind turbines; it primarily illustrates the relationship between the wind speed, pitch angle, and mechanical torque of a turbine rotor. By modeling the relationship between the wind speed and mechanical torque, the dynamic behavior of wind turbines can be more intuitively understood (Fig 1).

The mechanical torque generated by wind acting on the rotor of a DFIG can be determined as follows:


$$T_{wm}=\frac{\rho \pi R^{2}C_{P}V_{w}^{3}}{2\omega_{t}},$$ (1)

where *T*_*wm*_ is the mechanical torque experienced by the rotor due to wind and *Cp* is the wind energy conversion efficiency coefficient of the DFIG, which is expressed as follows:


$$\begin{array}{c} C_{p}=0.22\left(\frac{116}{\lambda_{i}}-0.4\beta -5\right)e^{\frac{12.5}{\lambda_{i}}}\\ \lambda_{i}=\frac{1}{\frac{1}{\lambda +0.08\beta }-\frac{0.035}{\beta^{3}+1}},\\ \lambda =\frac{\omega_{t}R}{V_{w}} \end{array}$$ (2)

where *λ* is the optimal tip speed ratio and *β* is the pitch angle.

The voltage and flux linkage equations of the DFIG in the *dq* coordinate system can be expressed as follows:


$$\{\begin{array}{c} {}*20lu_{qs}=R_{s}i_{qs}+p\psi_{qs}+\omega_{b}\psi_{ds}\\ u_{ds}=R_{s}i_{ds}+p\psi_{ds}-\omega_{b}\psi_{qs}\\ u_{qr}=R_{r}i_{qr}+p\psi_{qr}+s\omega_{b}\psi_{dr}\\ u_{dr}=R_{r}i_{dr}+p\psi_{dr}-s\omega_{b}\psi_{qr} \end{array},$$ (3)

$$\{\begin{array}{c} {}*20l\psi_{qs}=L_{s}i_{qs}+L_{m}i_{qr}\\ \psi_{ds}=L_{s}i_{ds}+L_{m}i_{dr}\\ \psi_{qr}=L_{r}i_{qr}+L_{m}i_{qs}\\ \psi_{dr}=L_{r}i_{dr}+L_{m}i_{ds} \end{array},$$ (4)

where *Ψ*_*ds*_ and *Ψ*_*qs*_ denote the d-axis and q-axis stator flux components, respectively (Wb); *Ψ*_*dr*_ and *Ψ*_*qr*_ denote the d-axis and q-axis rotor flux components, respectively (Wb); *u*_*ds*_ and *u*_*qs*_ denote the d-axis and q-axis stator voltage components, respectively; *i*_*ds*_ and *i*_*qs*_ denote the d-axis and q-axis stator current components, respectively; *u*_*dr*_ and *u*_*qr*_ denote the d-axis and q-axis rotor voltage components, respectively; and *i*_*dr*_ and *i*_*q*r_ denote the d-axis and q-axis rotor-side current components, respectively. *L*_*s*_ is the self-inductance of equivalent two-phase stator windings, *L*_*r*_ is the self-inductance of equivalent two-phase rotor windings, *L*_*m*_ is the mutual inductance between the stator and rotor windings, and *s* is the slip ratio.

The state-space equations can be expressed as follows:


$$\frac{d}{dt}\left[\begin{array}{c} {}*20ci_{qs}\\ i_{ds}\\ i_{qr}\\ i_{dr} \end{array}\right]=\frac{\omega_{b}}{L_{r}L_{s}-L_{m}^{2}}M\left[\begin{array}{c} {}*20li_{qs}\\ i_{ds}\\ i_{qr}\\ i_{dr} \end{array}\right]+\frac{\omega_{b}}{L_{r}L_{s}-L_{m}^{2}}N\omega_{r}+\frac{\omega_{b}}{L_{r}L_{s}-L_{m}^{2}}T\left[\begin{array}{c} {}*20cu_{qs}\\ u_{ds}\\ u_{qr}\\ u_{dr} \end{array}\right],$$ (5)

$$M=\left[\begin{array}{cccc} {}*20c-R_{s}L_{r} & L_{m}^{2}\left(1-\omega_{r0}\right)-L_{r}L_{s} & R_{r}L_{m} & -L_{r}L_{m}\omega_{r0}\\ L_{r}L_{s}-L_{m}^{2}\left(1-\omega_{r0}\right) & -R_{s}L_{r} & L_{r}L_{m}\omega_{r0} & R_{r}L_{m}\\ R_{s}L_{m} & L_{s}L_{m}\omega_{r0} & -R_{r}L_{s} & L_{m}^{2}-L_{r}L_{s}\left(1-\omega_{r0}\right)\\ -L_{s}L_{m}\omega_{r0} & R_{s}L_{m} & L_{r}L_{s}\left(1-\omega_{r0}\right)-L_{m}^{2} & -R_{r}L_{s} \end{array}\right],$$

$$N=\left[\begin{array}{c} -L_{\text{m}}^{2}i_{\text{ds}0}-L_{\text{r}}L_{\text{m}}i_{\text{dr}0}\\ L_{\text{m}}^{2}i_{\text{qs}0}+L_{\text{r}}L_{\text{m}}i_{\text{qr}0}\\ L_{\text{s}}L_{\text{m}}i_{\text{ds}0}+L_{\text{r}}L_{\text{s}}i_{\text{dr}0}\\ -L_{\text{s}}L_{\text{m}}i_{\text{qs}0}-L_{\text{r}}L_{\text{s}}i_{\text{qr}0} \end{array}\right]T=[\begin{array}{cccc} L_{\text{r}} & 0 & -L_{\text{m}} & 0\\ 0 & L_{\text{r}} & 0 & -L_{\text{m}}\\ -L_{\text{m}} & 0 & L_{\text{s}} & 0\\ 0 & -L_{\text{m}} & 0 & L_{\text{s}} \end{array}].$$

**Fig 2:**
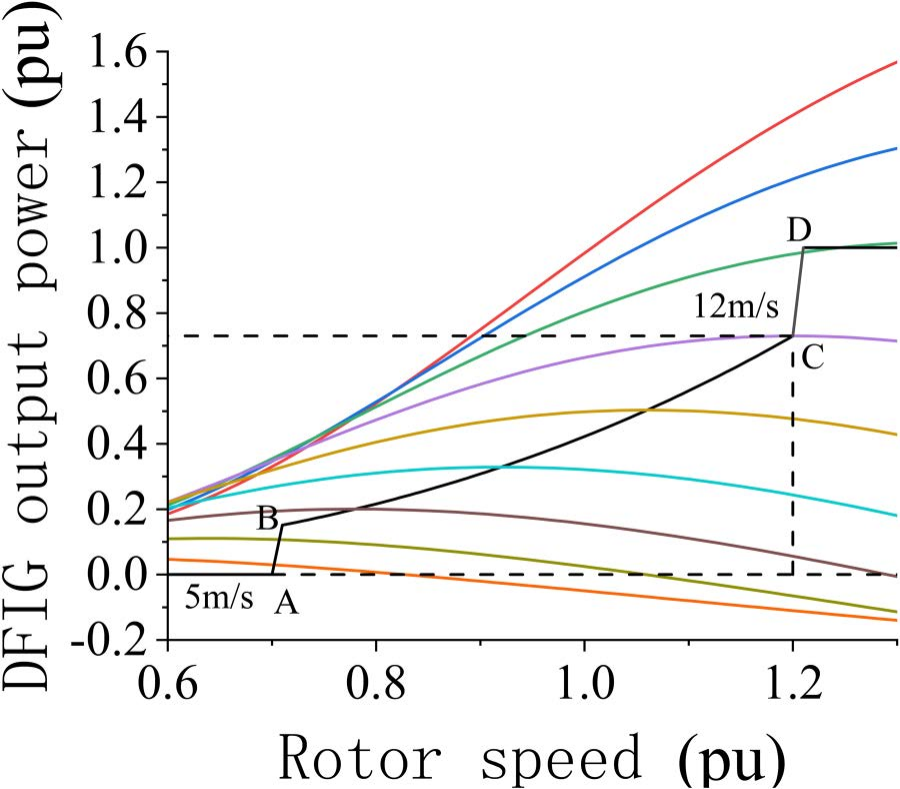
MPPT operating curve of the DFIG.

The DFIG features a unique maximum power point tracking (MPPT) characteristic and achieves optimized power output at various wind speeds. Its rotational speed can vary within 20%–30% above and below the synchronous speed; thus, it can adapt to wind-speed fluctuations and achieve improved power generation efficiency. By independently controlling the power output using rotor-side and grid-side converters, the DFIG controls the power factor and enhances grid compatibility. By managing the rotor-side converter in real time, the DFIG can show fast dynamic response capabilities and rapidly adjust the power output to ensure system stability and efficient operation. Fig 2 shows the MPPT operating curve of the DFIG.

The operational state of the DFIG can be divided into four stages based on variations in the wind speed, rotor speed, power output, and other factors. These stages and their characteristics and significance in wind power systems are discussed below:

1) Start-up stage: Under low-wind-speed conditions, the DFIG does not attain its minimum operating speed and hence cannot generate power. When the wind speed exceeds the cut-in wind speed, the DFIG begins to harvest energy. Moreover, the rotor speed gradually increases; thus, the DFIG transitions into the operational state. Segment AB in Fig 2 shows the gradual increase in the wind and rotor speeds until reaching the cut-in wind speed.
2) Subrated-wind-speed stage: Once the wind speed exceeds the cut-in value, the turbine adjusts its rotor speed to harvest maximum wind energy. In the “maximum power capture zone” (segment BC), the turbine adjusts its rotor speed based on variations in the wind speed and maintains an optimal value of the power coefficient (Cp) to maximize wind energy harvesting. As the wind speed continues to increase, the turbine transitions into the “constant rotor speed zone” (segment CD). In this zone, the rotor speed remains stable and adjusts the pitch angle to increase the mechanical torque, which gradually increases the output power toward the rated value.
3) Rated power stage: When the wind speed reaches the rated value, the DFIG adjusts the pitch angle to limit wind energy input. Thus, the DFIG maintains the rotor speed and power output within safe operating limits to prevent overloading and potential equipment damage. During this stage, the output power of the turbine remains steady at the rated value, ensuring a safe and stable operation.
4) Shut-down stage: When the wind speed exceeds the maximum rated wind speed of the turbine, the rotor speed and power cannot be controlled even by adjusting the pitch angle. Consequently, the turbine enters the shut-down state either by disconnecting from the grid or by turning off to avoid damage. It remains in this state until the wind speed decreases to a safe rated value.

The MPPT operating characteristic of the DFIG endows modern wind power systems with substantial economic and technical advantages. Through MPPT, the DFIG can harvest maximum power under varying wind-speed conditions, considerably improving the efficiency of wind power systems in harnessing wind energy.

## Rotor-side converter control strategy based on the improved NSGA-II

### Rotor-side converter working principle

The DFIG primarily comprises rotor-side and grid-side controllers that work together to establish a connection between the induction generator and grid. The rotor-side converter primarily regulates the power output of the generator via the decoupled control of d and q axes, enhancing the dynamic response of the system. By controlling the reactive power output, the rotor-side converter regulates the grid voltage and improves grid stability. As the extent of the grid integration of wind power increases, the converter design must meet higher efficiency and reliability standards. Under complex operating conditions, intelligent algorithms are required for optimizing the converter performance.

**Fig 3:**
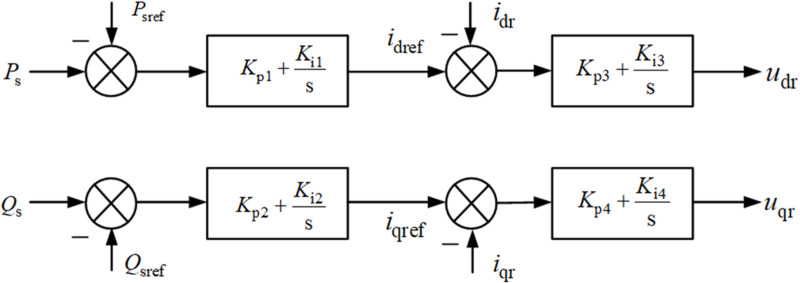
Control block diagram of the rotor-side controller based on stator voltage orientation.

Fig 3 shows the control block diagram of the rotor-side controller. *P*_*sref*_ and *Q*_*sref*_ denote the reference values of the active power and reactive power, respectively.

The relationship between the output power of the system and the dq-axis current of the rotor side can be expressed as follows:


$$\left\{ \begin{array}{c} P_{W}=\frac{L_{m}}{L_{s}}U_{s}i_{rd}\\ Q_{W}=-\frac{1}{L_{s}}y_{s}U_{s}-\frac{L_{m}}{L_{s}}u_{sd}i_{rq} \end{array}\right..$$ (6)

By adjusting the d- and q-axis currents of the rotor side, the output power of the DFIG can be controlled.


$$\left\{ \begin{array}{c} i_{rd,ref}=k_{p1}(P_{W,ref}-P_{W})+k_{i1}\int (P_{W,ref}-P_{W})dt\\ i_{rq,ref}=k_{p2}(Q_{W,ref}-Q_{W})+k_{i2}\int (Q_{W,ref}-Q_{W})dt \end{array}\right.,$$ (7)

where *i*_*rd,ref*_ and *i*_*rq,ref*_ represent the reference values of the d- and q-axis currents of the rotor side, respectively.

The state-space equation of the converter model is as follows:


$$\begin{array}{c} \frac{d}{dt}\left[\begin{array}{c} {}*20lx_{1}\\ x_{2}\\ x_{3}\\ x_{4} \end{array}\right]=\left[\begin{array}{cccc} {}*20c0 & 0 & 0 & 0\\ K_{i1} & 0 & 0 & 0\\ 0 & 0 & 0 & 0\\ 0 & 0 & K_{i2} & 0 \end{array}\right]\left[\begin{array}{c} {}*20cx_{1}\\ x_{2}\\ x_{3}\\ x_{4} \end{array}\right]+\left[\begin{array}{cccc} {}*20cu_{qs0} & u_{ds0} & 0 & 0\\ K_{p1}u_{qs0} & K_{p1}u_{qs0} & 0 & -1\\ u_{ds0} & -u_{qs0} & 0 & 0\\ K_{p2}u_{qs0} & -K_{p2}u_{qs0} & -1 & 0 \end{array}\right]\left[\begin{array}{c} {}*20ci_{qs}\\ i_{ds}\\ i_{qr}\\ i_{dr} \end{array}\right]+\\ \left[\begin{array}{cccc} {}*20ci_{qs0} & i_{ds0} & 0 & 0\\ K_{p1}i_{qs0} & K_{p1}i_{ds0} & 0 & 0\\ -i_{ds0} & i_{qs0} & 0 & 0\\ -K_{p2}i_{qs0} & K_{p2}i_{qs0} & 0 & 0 \end{array}\right]\left[\begin{array}{c} {}*20lu_{qs}\\ u_{ds}\\ u_{qr}\\ u_{dr} \end{array}\right] \end{array}$$ (8)

### Control optimization algorithm and improvement strategy

The NSGA-II is a flexible and powerful optimization tool; it is particularly highly suitable for solving problems that cannot be addressed using traditional optimization methods. Although it has a low computational efficiency and requires parameter tuning, the NSGA-II can generate effective solutions in many complex scenarios with appropriate algorithm design and optimization. Because of the nondominated sorting approach, the NSGA-II exhibits excellent performance when handling large-scale problems and reduces computational complexity. By introducing crowding distance, it maintains solution diversity and ensures that the Pareto front covers maximum potential solutions. In complex optimization problems requiring a comprehensive trade-off among multiple objectives, the NSGA-II provides decision makers with numerous alternatives.

When optimizing the rotor-side controller parameters of the DFIG using the NSGA-II, the algorithm must be integrated with objective functions to simultaneously minimize the rotor-side current and reduce total harmonic distortion (THD). The NSGA-II excels in multiobjective optimization; however, it has some limitations. With increasing number of objectives, the computational cost of the NSGA-II considerably increases. It may also encounter resource bottlenecks when handling high-dimensional multiobjective problems. To address the limitations of the traditional NSGA-II algorithm in handling complex, multiobjective control parameter optimization problems for DFIGs, this study introduces a set of novel strategies that considerably improve the algorithm’s global search capability, convergence speed, and robustness against premature convergence. In particular, two mechanisms are integrated into the NSGA-II framework: a knight elite strategy and an automatic parameter tuning procedure, both of which are tailored for the dynamic control environment of wind turbine systems.

The knight elite strategy, introduced during the population update phase, enhances the diversity and exploratory capabilities of the population. The control parameter space (in particular, the proportional and integral gains, *K*_*p*_ and *K*_*i*_, respectively) is divided into three equal subintervals along each dimension. In each subinterval, candidate solutions are randomly generated, resulting in nine individuals representing different regions of the solution space. This targeted diversification helps mitigate the risk of premature convergence by ensuring broad coverage of the parameter domain, which is critical in highly nonlinear, constrained environments, such as those experienced during grid voltage disturbances. To the best of our knowledge, such a structured segment-based update mechanism has not yet been employed for the NSGA-II or wind-power controller optimization problems. To address this research gap, we introduce a two-stage automatic parameter tuning process that refines the search granularity to further improve the solution quality. In the first stage, the integer part of each parameter is adjusted based on fitness trends, thereby narrowing down the search space to a more promising region. In the second stage, the decimal portion is fine-tuned using a bidirectional local search strategy. In particular, upward and downward decimal searches are performed in the ± 1 range of the selected integer values. This hierarchical search approach simplifies the optimization process while maintaining high precision in locating the optimal solution. It also reduces computational overhead by avoiding the redundant evaluation of low-quality parameter regions. The aforementioned two mechanisms work synergistically: the knight elite strategy ensures effective exploration across the global parameter space, while the automatic tuning process enhances local exploitation around the candidate optima. Thus, these mechanisms enable the improved NSGA-II to achieve a better balance between convergence speed and solution diversity, resulting in significantly more reliable and accurate control parameters for DFIG systems under transient fault conditions. In addition, by explicitly targeting key engineering objectives, such as minimizing rotor-side current overshoot, reducing harmonic distortion, and shortening fault recovery time, the improved algorithm goes beyond theoretical optimization and delivers practical benefits in real-world wind energy applications.

The initial population size N is determined first, and the upper and lower bounds of the optimization parameters are set based on system stability:


$$\begin{array}{c} P=(K_{p},K_{i})\\ K_{p\min}\leq K_{p}\leq K_{p\max},\\ K_{i\min}\leq K_{i}\leq K_{i\max} \end{array}$$ (9)

Based on the operational stability of the system, the parameter values shown in Table 1 are determined.

**Table 1:** Parameter values.

Parameter	Value
*K*_*pmin*_	0.005
*K*_*pmax*_	2
*K*_*imin*_	0.5
*K*_*imax*_	5

In the proposed knight elite strategy, the parameter spaces of the proportional and integral gains (*K*_*p*_ and *K*_*i*_, respectively) are each uniformly divided into three equal-length subintervals.


$$\begin{array}{c} K_{p}(K_{p,\min},K_{p,1})\;(K_{p,1},K_{p,2})\;(K_{p,2},K_{p,\max})\\ K_{i}\;(K_{i,\min},K_{i,1})\;(K_{i,1},K_{i,2})\;(K_{i,2},K_{i,\max})\\ K_{p,1}=K_{p,\min}+\frac{1}{3}(K_{p,\max}-K_{p,\min})\\ K_{p,2}=K_{p,\min}+\frac{2}{3}(K_{p,\max}-K_{p,\min})\\ K_{i,1}=K_{i,\min}+\frac{1}{3}(K_{i,\max}-K_{i,\min})\\ K_{i2}=K_{i,\min}+\frac{2}{3}(K_{i,\max}-K_{i,\min}) \end{array}$$ (10)

To generate diverse and representative candidate solutions across the search space, a new individual is randomly sampled from each subregion formed by the Cartesian product of the following intervals:


$$[{K_{p}}^{i,j},{K_{i}}^{i,j};\sim \;u[{K_{p}}^{i}]\times u[{K_{i}}^{j}],$$ (11)

where *u* denotes a uniform distribution within the specified interval. This results in 3 × 3 (i.e., 9) new individuals, each sampled from a distinct region of the parameter space. This structured coverage improves global exploration and mitigates premature convergence.

The improved optimization strategy first tunes the integer part of the parameter, followed by fine-tuning the decimal part. It first searches for an optimal integer part. After the preliminary values for *K*_*p*_ and *K*_*i*_ are obtained, the decimal part is optimized. An upward and downward search strategy is incorporated, which expands the search space to


$$(K_{p},K_{i})\in [K_{p}*-1,K_{p}*+1]\times [K_{i}*-1,K_{i}*+1].$$ (12)

The optimization range for the decimal part is


$$\begin{array}{c} {}[K_{p}*+m,K_{i}*+nnonumber\\ m,n\in [-0.99,0.99] \end{array}$$ (13)

Two strategies can be used for optimizing the decimal part:

(i) search with a fixed step size in the upward direction


$$\begin{array}{c} K_{p}=K_{p}*+m,K_{i}=K_{i}*+n,\\ m,n\in [0,0.99],\\ m,n=-k\Delta ,k=1,2,... \end{array}$$ (14)

or (ii) search with a fixed step size in the downward direction


$$\begin{array}{c} K_{p}=K_{p}*+m,K_{i}=K_{i}*+n,\\ m,n\in [-0.99,0],\\ m,n=-k\Delta ,k=1,2,... \end{array}$$ (15)

The results of the integer- and decimal-part optimization stages are then combined to obtain the final solution.

The objective function is calculated as follows:


$$\begin{array}{c} f_{1}=i_{r}=sum({\mathrm{idr}}^{\wedge }2)+sum({\mathrm{idq}}^{\wedge }2)\\ f_{2}=THD=\frac{\sqrt{\sum_{n=2}^{N}I_{n}^{2}}}{I_{1}} \end{array}$$ (16)

Here, the goal is to minimize *i*_r_ and THD to the maximum extent possible. Next, the population is sorted based on the nondomination levels. The conditions for combining *K*_*p1*_ and *K*_*i1*_ that dominate *K*_*p2*_ and *K*_*i2*_ are as follows:


$$f_{1}(K_{p1},K_{i1})\leq f_{1}(K_{p2},K_{i2}),f_{2}(K_{p1},K_{i1})\leq f_{2}(K_{p2},K_{i2}).$$ (17)

The dominating combinations *n*_*p*_ and *S*_*p*_ can be calculated as follows:


$$\begin{array}{c} n_{p}=\{q\in P,\;qdominates\mathrm{\backslash nolimits}p\}\\ S_{p}=\{q\in P,\;pdominates\mathrm{\backslash nolimits}q\} \end{array}$$ (18)

The first layer of nondominated solutions is as follows:


$$F_{1}=\{p\in P,\;n_{p}=0\}.$$ (19)

The remaining layers are then divided based on dominant relationships.


$$F_{i+1}=\{q\in S_{p}:\forall p\in F_{i},n_{q}=n_{q}-1,n_{q}=0\}.$$ (20)

Subsequently, the nondominant sorting of population is performed.


$$F_{1}\subset F_{2}\subset ...\subset F_{n}.$$ (21)

Here, *F*_*i*_ denotes the nondominated solution at the *i*_*th*_ layer.

The crowding distance of each individual solution is computed as follows:


$$d(i)=\frac{f_{1}^{i+1}-f_{1}^{i-1}}{\max f_{1}-\min f_{1}}+\frac{f_{2}^{i+1}-f_{2}^{i-1}}{\max f_{2}-\min f_{2}}$$ (22)

Here, $f_{1}^{i+1}$ and $f_{1}^{i-1}$ represent the objective function values. Next, the adjacent solution values are compared.

The simulated binary crossover (SBX) and polynomial mutation operators are used to generate new solutions.


$$(K_{p},K_{i}{)}^{\prime }=(K_{p},K_{i})+\eta ((K_{p},K_{i}{)}_{\max}-(K_{p},K_{i}{)}_{\min}).$$ (23)

**Fig 4:**
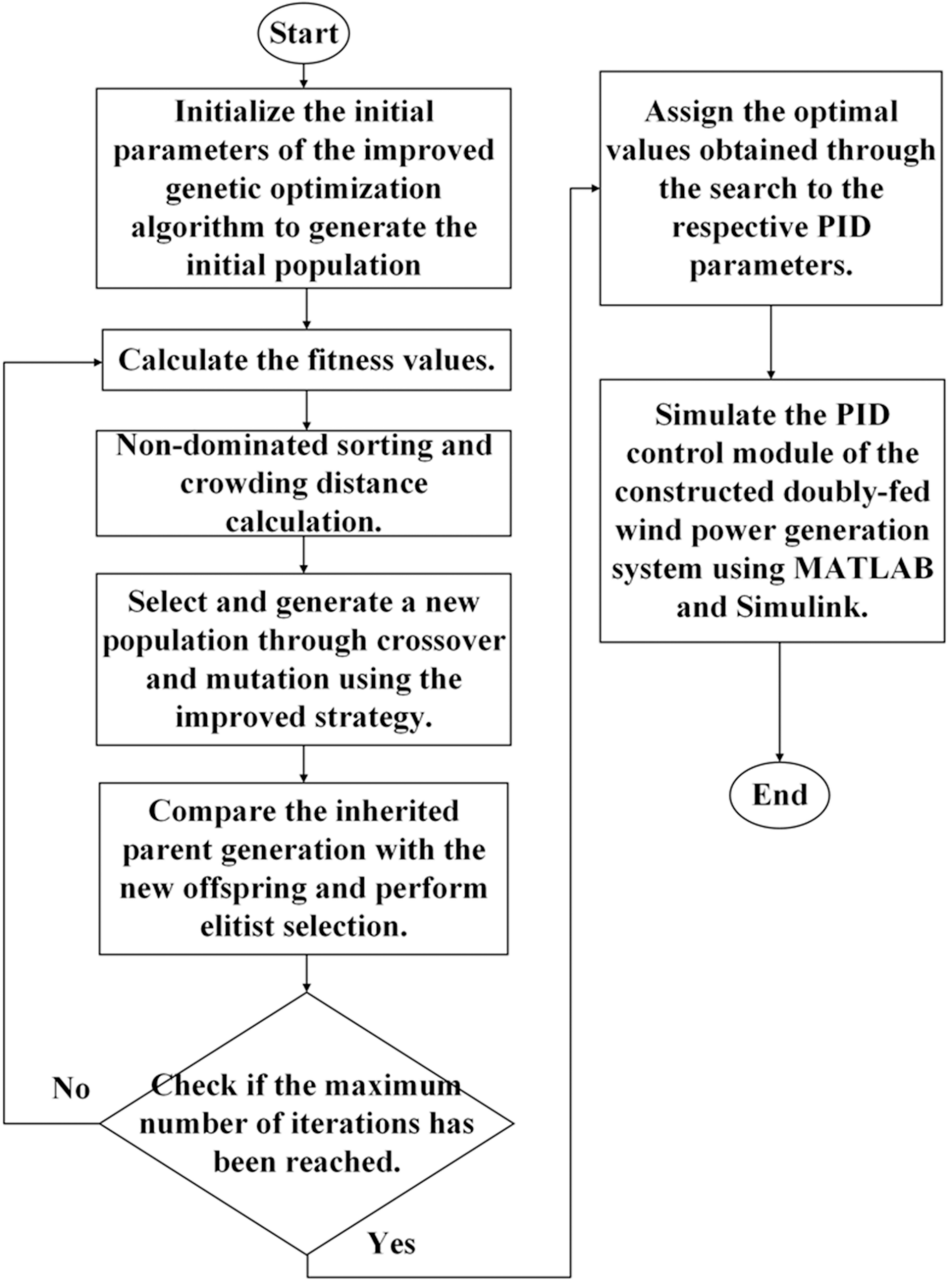
Optimization of DFIG PID controller parameters using the improved NSGA-II.

Furthermore, the parent and offspring populations are merged, and the next generation is selected based on the nondomination levels and the crowding distance. The results are output when the optimization process reaches the maximum number of iterations (Fig 4).

## Simulation analysis

Rational parameter optimization reduces the effect of DC components and AC decay components as well as reduces the duration of overcurrent events. Consequently, the equipment stress decreases and the lifespan of the components increases. A multiobjective optimization method and simulation analysis are required to determine the optimal parameter combination for tuning a PID controller. Traditional PID controller parameters are typically set using empirical formulas or simple tuning methods; optimal results cannot be easily obtained for complex nonlinear systems by employing these formulas and methods. When dealing with HVRT issues in wind turbines, appropriate PID controller parameter settings are crucial for reducing the peak current of the rotor-side converter. Hence, a multiobjective optimization method based on an improved NSGA-II is proposed herein for optimizing PID controller parameters. This method simultaneously reduces current surges at the rotor-side converter and optimizes the dynamic response characteristics of the system. To verify the effectiveness of the proposed method, a system model is developed in MATLAB/Simulink for simulation analysis.

Before analyzing the convergence behavior of the system output, we define the applied disturbances and key parameters of the DFIG. The parameters and their values are summarized in Table 2.

**Table 2:** Key parameters of DFIG.

Parameter	Value
Rated capacity	1.5 MW
Stator reactance	0.08 pu
Rated voltage	0.69 kV
Rotor reactance	0.09 pu
Rated frequency	50 Hz
Mutual reactance	2.75 pu
Stator resistance	0.69 kV
DC capacitor	0.09 pu
Rotor resistance	50 Hz
DC bus-rated voltage	2.75 pu

**Fig 5:**
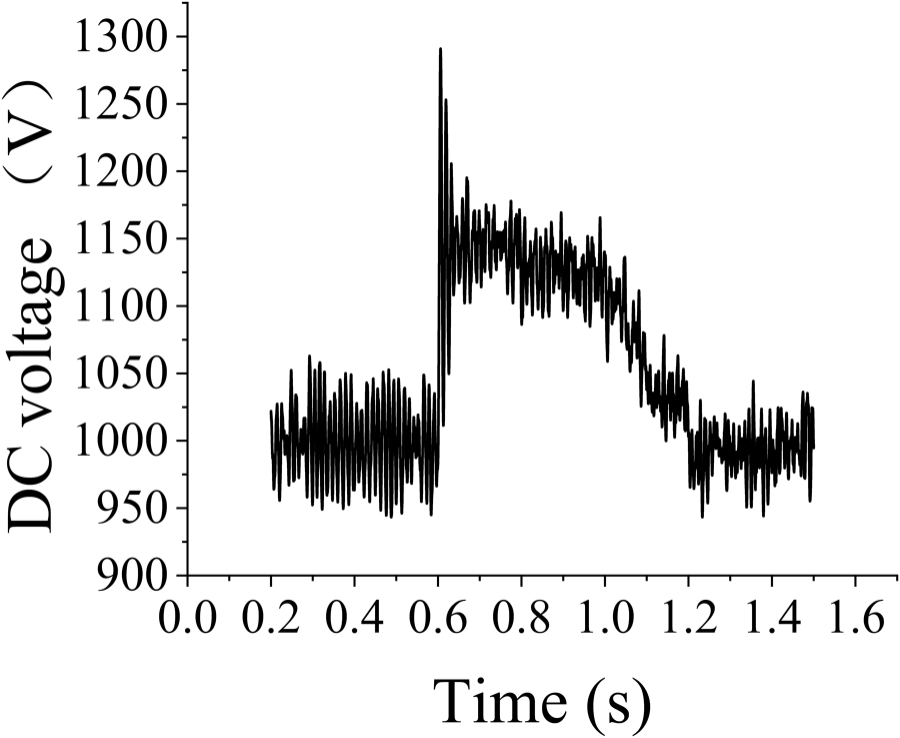
DC bus voltage variations.

**Fig 6:**
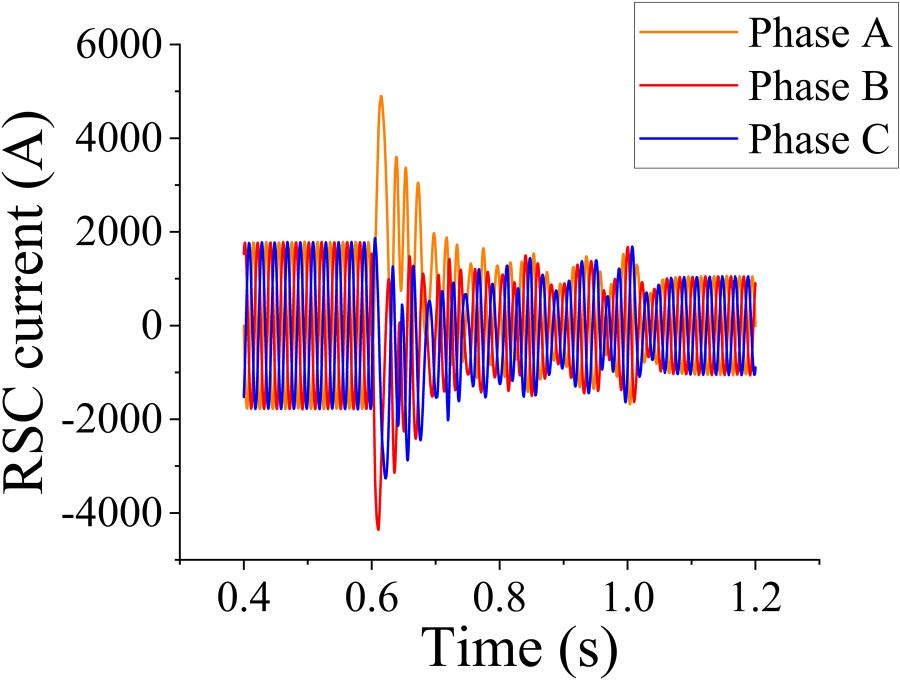
Rotor-side three-phase current variation.

Fig 5 illustrates the behavior of the DC bus voltage during a 0.6-s HVRT event. The fault occurs for 0.4 s when the grid-side voltage increases to 1.1 times the nominal value. Prior to the fault, the DC bus voltage remains stable, reflecting the system’s normal operational state. Following the voltage surge, the DC bus voltage rapidly recovers and stabilizes, indicating that the DFIG operates within its normal operating conditions, effectively maintaining voltage stability during grid disturbances. The sampling step size used in the simulation is 1e-05 s. Fig 6 shows the three-phase current of the rotor-side converter during the HVRT event. Initially, the three-phase current exhibits a transient increase in response to the grid-voltage surge, reflecting the system’s dynamic response to the disturbance. This increase is followed by a gradual stabilization of the current, where the system settles back into a steady-state operating condition. The observed transient behavior of the current corresponds to the converter’s active regulation of power output during the voltage fluctuation. The two figures confirm the optimal operation of the DFIG model. The smooth transition of the current to a steady state further highlights the effective control strategy employed for the rotor-side converter, demonstrating that the Simulink model continues to operate under normal, stable working conditions throughout the HVRT event.

**Fig 7:**
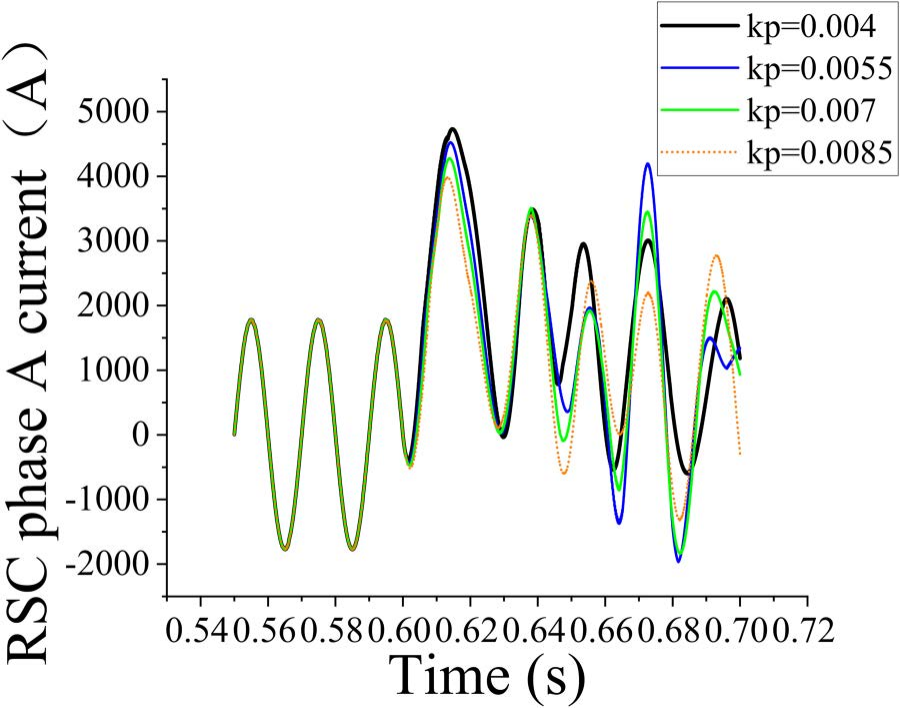
Effect of different *K*_*p*_ values on the rotor-side current with *K*_*i* _ = 1.

**Fig 8:**
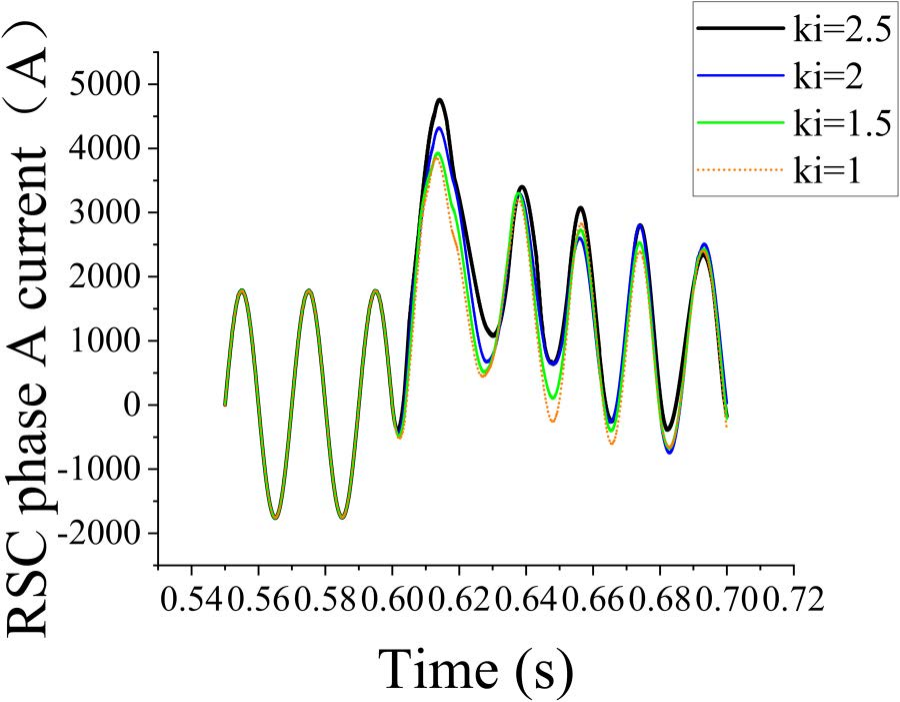
Effect of different *K*_*i*_ values on the rotor-side current with *K*_*p*_  = 0.008.

In the PID loop, the first set data set was obtained by setting *K*_*i* _= 1 and *K*_*p*_ = 0.004, 0.0055, 0.007, and 0.0085; the corresponding simulation results are shown in Fig 7. The second data set was obtained by setting *K*_*p*_ = 0.0085 and *K*_*i*_ = 1, 1.5, 2, and 2.5; the corresponding simulation results are shown in Fig 8.

The aforementioned simulation results reveal that different combinations of *K*_*p*_ and *K*_*i*_ considerably affect the system performance, indicating that parameter optimization can substantially improve the system performance. The analysis of simulation waveforms revealed current peak variations and the overall stability and harmonic characteristics of the system. To further understand the effect of *K*_*p*_ and *K*_*i*_ values on current peaks, the simulation data were quantitatively analyzed. The waveforms for each combination of *K*_*p*_ and *K*_*i*_ reflected the dynamic response of the system and its steady-state performance under each set parameter combination. Comparative data analysis revealed that considerably high and low parameter values increased the current peaks and that the current peaks were minimized within a certain parameter range. This result provided important theoretical support for subsequent parameter tuning.

**Fig 9:**
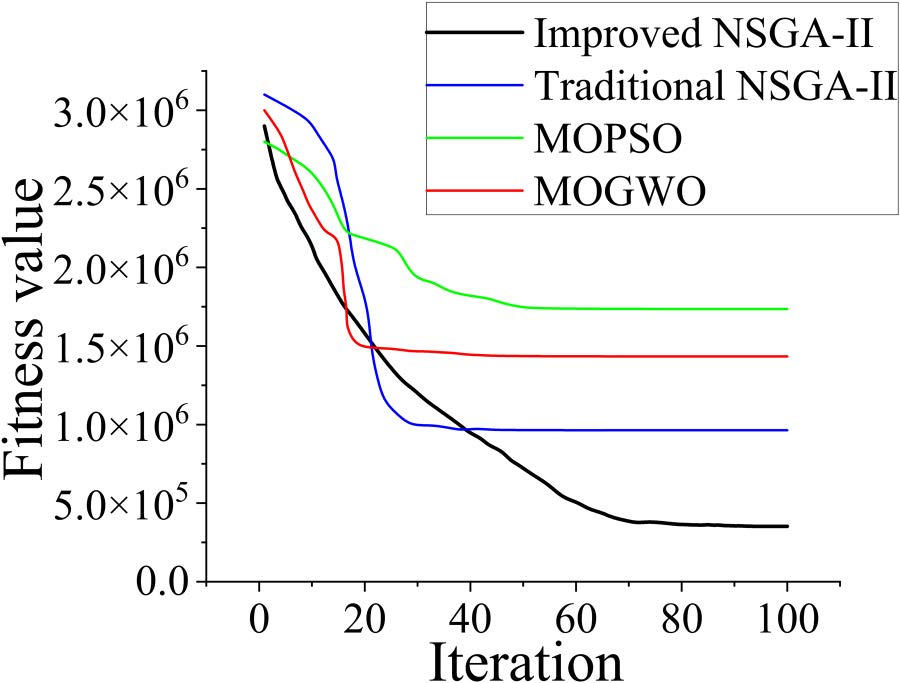
Fitness value convergence of the four optimization algorithms.

Table 3 presents a comparison of the improved NSGA-II, traditional NSGA-II, MOPSO algorithm, and MOGWO algorithm by showcasing the optimized PID controller parameters obtained using these algorithms. Additionally, the convergence of the fitness values for each algorithm is illustrated in Fig 9, which shows how the fitness values evolve over generations.

**Table 3:** Optimized PID controller parameters obtained using the four optimization algorithms.

Parameter	Traditional NSGA-II	Improved NSGA-II	MOPSO	MOGWO
*K*_p_	0.00932	0.00793	0.01304	0.01009
*K*_i_	1.54621	1.01171	1.92534	1.78623

In this study, we performed a comprehensive computational complexity analysis of four optimization algorithms (improved NSGA-II, traditional NSGA-II, MOPSO algorithm, and MOGWO algorithm) to evaluate their computational efficiency and scalability, particularly in high-dimensional optimization problems and multi-DFIG systems. The improved NSGA-II exhibited the highest computational efficiency. Its time complexity was dominated by the nondominated sorting step, with a time complexity of O(P^2^), where P is the population size. Despite this quadratic time complexity, the improved NSGA-II remained more efficient than the other algorithms, particularly in terms of robustness and convergence speed. This advantage can be attributed to its ability to solve multiobjective optimization problems effectively while maintaining a low computational burden. The overall time complexity per generation for the improved NSGA-II was O(G ⋅ P^2^), while the space complexity for this algorithm was O(P ⋅ d), where d is the number of decision variables. The traditional NSGA-II exhibited efficiency close to that of the improved NSGA-II. Although the overall time complexity for the traditional NSGA-II was also O(G ⋅ P^2^), its convergence was slower than that of the improved NSGA-II in some cases. Nevertheless, it is a reliable choice for multiobjective optimization. Similar to the improved NSGA-II, its space complexity is O(P ⋅ d).

Compared with the traditional and improved NSGA-II, the MOPSO and MOGWO algorithms a exhibited a lower optimal performance. Both algorithms demonstrated linear growth in computational complexity with time complexities of O(G ⋅ P ⋅ d), which is higher than the quadratic time complexity of the traditional and improved NSGA-II. The increased dimensionality and the required particle and wolf position updates contributed to the higher computational burden of the MOPSO and MOGWO algorithms. Thus, the MOPSO and MOGWO algorithms required more computational resources, particularly for problems with higher dimensions or larger population sizes. The space complexity for both algorithms was O(P ⋅ d), which is consistent with that of the traditional and improved NSGA-II. In quantitative experiments involving a population size of 100, 100 generations, and a 10-dimensional problem, the improved NSGA-II completed the optimization process in 23.2 s. The traditional NSGA-II completed the optimization process slightly faster (21.3 s) than the improved algorithm but at the expense of the robustness of the optimization results. The MOPSO and MOGWO algorithms completed the optimization process faster than both the NSGA-II versions, with run times of 18.5 and 20.9 s, respectively; however, the quality of their optimization results was lower than that of the improved NSGA-II.

The improved NSGA-II outperformed all the other algorithms in terms of solution quality, exhibiting enhanced convergence and robustness. Although it requires more computational resources than the traditional NSGA-II, it achieves better results in complex optimization tasks. The outstanding performance of the improved NSGA-II algorithm in terms of its solution quality and robustness enables it to effectively handle the complexities of multi-DFIG systems and high-dimensional optimization tasks. In addition, its enhanced convergence properties and adaptive mechanisms allow it to efficiently optimize the control parameters of each turbine while maintaining the overall system stability. The ability to balance exploration and exploitation ensures that the improved algorithm can be effectively scaled to handle the increased complexities associated with large systems. Thus, the improved NSGA-II algorithm is well-suited for real-world applications where the system size and optimization complexity continuously increase.

**Fig 10:**
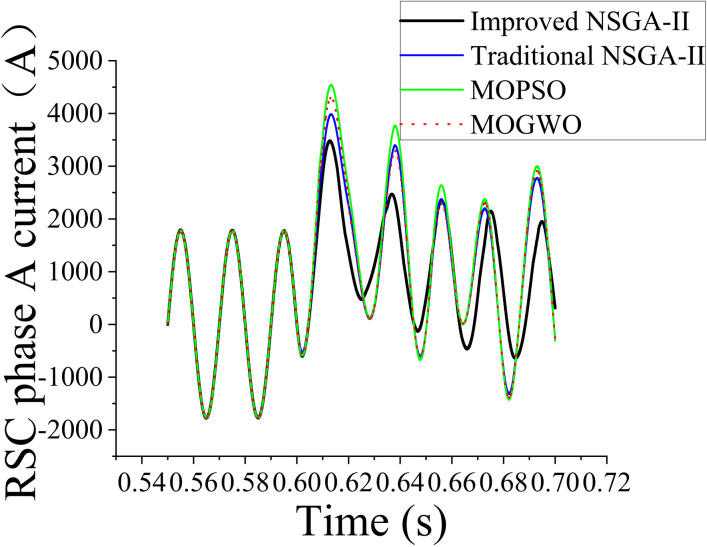
Comparison of the rotor-side phase-A current at 1.2 times the nominal voltage after optimization using the different algorithms.

To validate the superiority of the proposed algorithm, its optimization results were compared with those of the traditional NSGA-II, MOPSO algorithm, and MOGWO algorithm. The optimized PID parameters obtained using each algorithm were implemented in Simulink simulations under a grid fault scenario with a grid voltage 1.2 times the nominal value as well as a fault occurring at 0.6 s and lasting for 0.4 s. The actual effect of the optimized parameters on the system performance was evaluated via comparative analysis; the results are shown in Fig 10 (rotor-side phase-A current waveform). Four key performance metrics were compared: voltage response after boost (first and second cycles), iteration counts required to achieve the optimal solution, and fitness of the obtained optimal results.

The voltage-boost response provides critical insights into the dynamic performance of each optimization method in regulating the DFIG system. After the first voltage-boost cycle, the improved NSGA-II achieved the lowest current value (3484.79077 A), indicating superior stability and reduced transient oscillations compared with the traditional NSGA-II (3965.60702 A), MOPSO algorithm (4520.79200 A), and MOGWO algorithm (4282.85558 A). After the second voltage-boost cycle, the performance gap became even more pronounced. The improved NSGA-II demonstrated a substantial current reduction to 2473.26821 A, while the traditional NSGA-II showed a current of 3295.61013 A, the MOPSO algorithm showed a current of 3771.2652 A, and the MOGWO algorithm showed a current of 3397.53622 A. This indicated that the improved NSGA-II ensured a faster and more stable voltage regulation process, highlighting its effectiveness in dynamic control scenarios. The traditional NSGA-II required 35 iterations to identify the optimal solution, while the improved NSGA-II required 79 iterations. Although the iteration count was higher for the latter algorithm, it indicated a more thorough search process, which led to a better solution. Furthermore, the MOPSO (45 iterations) and MOGWO (21 iterations) algorithms exhibited faster convergence compared with the improved NSGA-II. However, this result is not necessarily advantageous as it may result in suboptimal solutions due to premature convergence. The fitness value provides a direct measure of the optimization quality. A lower fitness value generally corresponds to a better solution in the context of this optimization problem. The improved NSGA-II achieved the lowest fitness value of 352215.6, signifying its ability to identify a more optimal controller parameter set than the traditional NSGA-II (964019.4), MOPSO algorithm (1735103), and MOGWO algorithm (1433592). The significantly low fitness value of the improved NSGA-II suggested that the proposed improvements to traditional NSGA-II enabled better exploration of the search space and the avoidance of local optima, leading to enhanced control performance.

To further investigate the system response under increased grid voltages, we considered a voltage boost of 1.3 times the nominal grid voltage, with a fault occurring at 0.6 s and lasting for 0.4 s. This analysis was similar to the previous one wherein (i) the voltage was 1.2 times the nominal grid voltage and (ii) the current responses in the first and second cycles after the voltage boost were recorded. The data recorded for the scenario with a voltage 1.3 times the nominal voltage captured transient current behaviors, enabling a more comprehensive assessment of the dynamic characteristics of the system. These results are essential for understanding the limits of operational stability and optimizing system performance under extreme voltage fluctuations.

**Fig 11:**
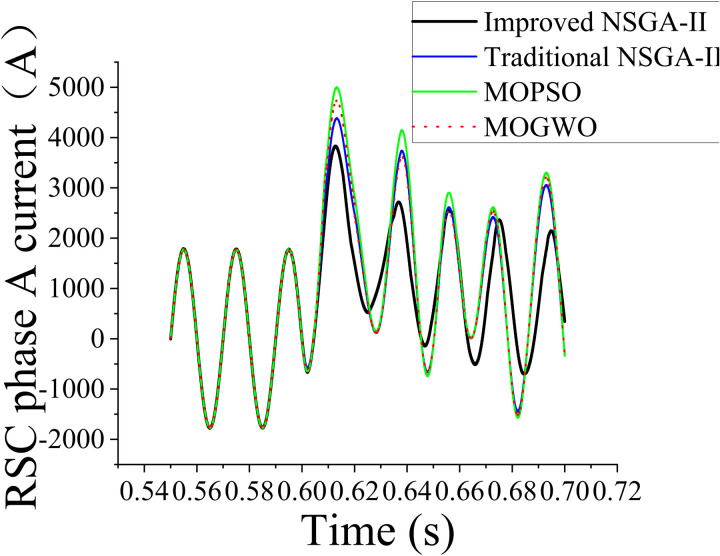
Current comparison at 1.3 times the nominal voltage boost after parameter optimization using the different algorithms.

The simulation waveforms shown in Figs. 10 and 11 and the comparison of current peaks listed in Tables 4 and 5 clearly demonstrate that when the parameters were optimized using the improved NSGA-II, highly stable current waveforms were observed during load variations. Furthermore, current peak values were reduced and subsequent secondary peaks were not observed. This indicated that the optimized parameters improved the dynamic response of the system, enabling it to recover more quickly and smoothly to steady-state conditions, particularly under load fluctuations and other disturbances. The optimized parameters effectively suppressed excessive current transients resulting from external disturbances, preventing system instability. Reduction of the current peaks improved the dynamic response of the system, enhanced the operational reliability and lifespan of the equipment, and reduced the risk of equipment failure due to overheating and excessive currents. The harmonic components in the current waveforms were also effectively suppressed, making the current waveforms similar to an ideal sine wave. This increased the operating stability and longevity of the equipment. Harmonic components degrade the power output quality of a system and may induce mechanical vibrations, increasing noise and wear. Current waveforms obtained using optimized parameters identified using the traditional algorithm showed considerable oscillations or irregular fluctuations that decayed slowly within a short time, indicating potential system instability. In practical operation, such an instability can lead to equipment overload. The improved NSGA-II can be applied to the DFIG drive system and extended to other types of converter control systems. In scenarios requiring high-precision control and enhanced system performance, similar optimization strategies can be used. Thus, we demonstrated that in practical applications, the proposed parameter optimization framework is well-suited for complex multiobjective control problems in hybrid AC/DC microgrid environments as it effectively enhances system stability, robustness, and overall performance.

**Table 4:** Performance comparison of the optimization algorithms under voltage boost to 1.2 times the nominal value.

Grid voltage boosted to 1.2 times the nominal value	Traditional NSGA-II	Improved NSGA-II	MOPSO	MOGWO
Current after voltage boost (first cycle)	3965.60702	3484.79077	4520.79200	4282.85558
Current after voltage boost (second cycle)	3295.61013	2473.26821	3771.2652	3397.53622
Iterations required for identifying the optimal solution	35	79	45	21
Fitness value of the optimal solution	964019.4	352215.6	1735103.0	1433592.0

**Table 5:** Performance comparison of the optimization algorithms under voltage boost to 1.3 times the nominal value.

Grid voltage boosted to 1.3 times the nominal value	Traditional NSGA-II	Improved NSGA-II	MOPSO	MOGWO
Current after voltage boost (first cycle)	4362.16772	3833.26984	4972.8712	4711.14113
Current after voltage boost (second cycle)	3625.17114	2720.59503	4148.3917	3737.28984

Herein, we achieved highly accurate identification results in the parameter optimization of a DFIG in a hybrid AC/DC microgrid environment. Based on these results, in our future research, we will focus on optimizing these parameters to enhance system stability, efficiency, and robustness under varying grid conditions. This optimization process will integrate advanced multiobjective evolutionary algorithms and real-time hardware-in-the-loop (HIL) validation to ensure practical feasibility and effectiveness. Including renewable energy forecasting techniques, such as wind-speed prediction and adaptive control strategies, will further improve the dynamic performance of the system, contributing to the reliable and intelligent operation of hybrid microgrid systems.

## Conclusions

Herein, a parameter optimization strategy for the rotor-side controller of a DFIG is proposed based on an improved NSGA-II. The optimized parameters were input into a MATLAB/Simulink model for simulation analysis, validating the effectiveness of the proposed control strategy. The key conclusions are as follows:

(1) By modifying the traditional NSGA-II via automatic parameter tuning and the knight elite strategy, its convergence speed was considerably increased. Although the improved NSGA-II required more iterations (79) than the traditional NSGA-II (35), its ability to explore the search space more thoroughly led to its significantly lower fitness value (352215.6) compared with those of the traditional NSGA-II (964019.4), the MOPSO algorithm (1735103), and MOGWO algorithm (1433592). This demonstrated that the improved algorithm effectively avoided local optima and identified optimized controller parameters for improved system performance.
(2) The improved NSGA-II considerably enhanced the stability and transient response of a DFIG system. After a voltage boost to 1.2 times the nominal value, the peak current decreased to 3484.79077 when using the proposed algorithm. Moreover, the corresponding values were 3965.60702, 4520.792, and 4282.85558 for the traditional NSGA-II, MOPSO algorithm, and MOGWO algorithm, respectively. After a voltage boost to 1.3 times the nominal value, the peak current decreased to 3833.26984 when using the improved NSGA-II. Additionally, the corresponding values were 4362.16772, 4972.8712, and 4711.14113 for the traditional NSGA-II, MOPSO algorithm, and MOGWO algorithm, respectively. This led to minimized current surges and improved overall system reliability. Furthermore, the optimized parameters effectively suppressed harmonic distortions, ensuring a smoother and more stable power output.
(3) The proposed optimization framework provides a reliable solution for improving DFIG performance and can be extended to other converter-controlled renewable energy systems. However, the proposed algorithm was validated exclusively through simulations without real-time experiments or hardware-based verification. Consequently, practical considerations, such as hardware constraints, real-time controller latency, and unforeseen disturbances, were not explicitly evaluated. In future research, we will perform real-time HIL validation to address these limitations and confirm the algorithm’s robustness and feasibility under dynamic and realistic grid conditions. Furthermore, integrating renewable energy forecasting techniques, such as wind-speed prediction and adaptive control, will enhance system resilience and operational efficiency in hybrid AC/DC microgrid environments. These steps will ensure a closer alignment between theoretical developments and practical engineering applications.

## Supporting information

S1 Text Additional references. (DOCX)

## Abbreviations

NSGA-II: Nondominated Sorting Genetic Algorithm II
DFIG: Doubly Fed Induction Generator
LVRT: Low-Voltage Ride-Through
HVRT: High-Voltage Ride-Through
MOPSO: Multiobjective Particle Swarm Optimization
MOGWO: Multiobjective Gray Wolf Optimization
PID: Proportional–Integral–Derivative.
