# Supplementary material for: Optimization of rotor-side controller parameters in doubly fed induction generators based on an improved NSGA-II
Source: PLoS One. 2025 Jun 23;20(6):e0326077. doi: 10.1371/journal.pone.0326077 (PMC12185016; doi:10.1371/journal.pone.0326077)
Supplement: S1 Text — (DOCX) [file pone.0326077.s001.docx]

**Supporting Information**

S1 Fig. Structure of the DFIG


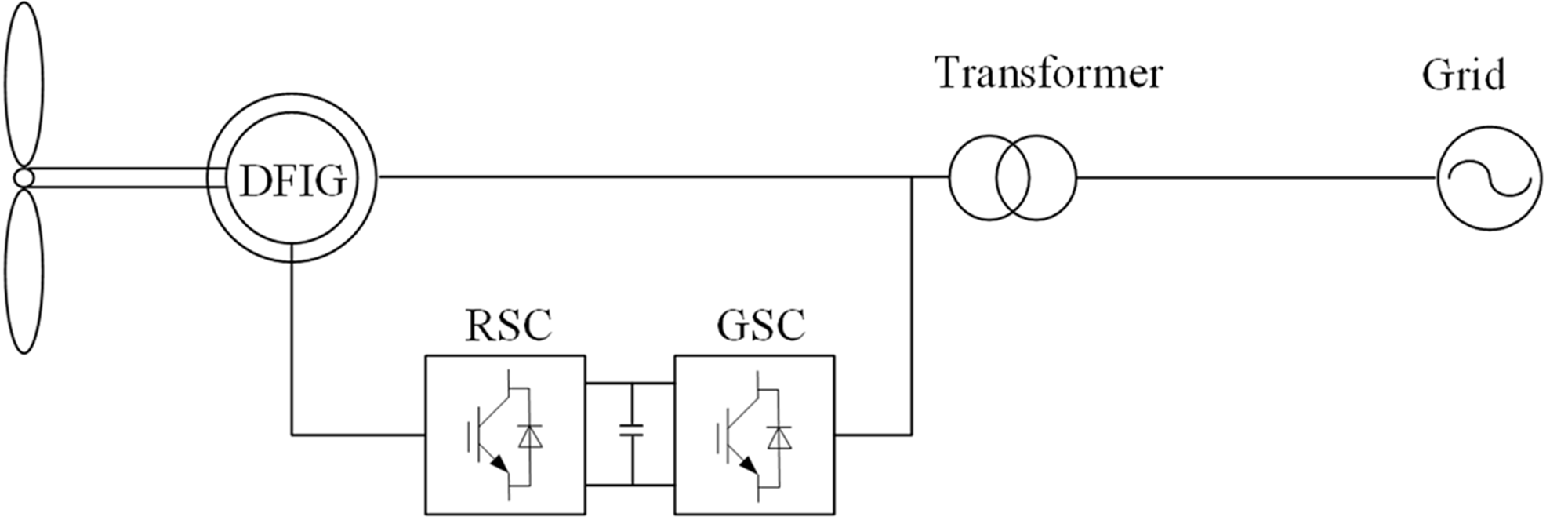


S2 Fig. MPPT operating curve of the DFIG


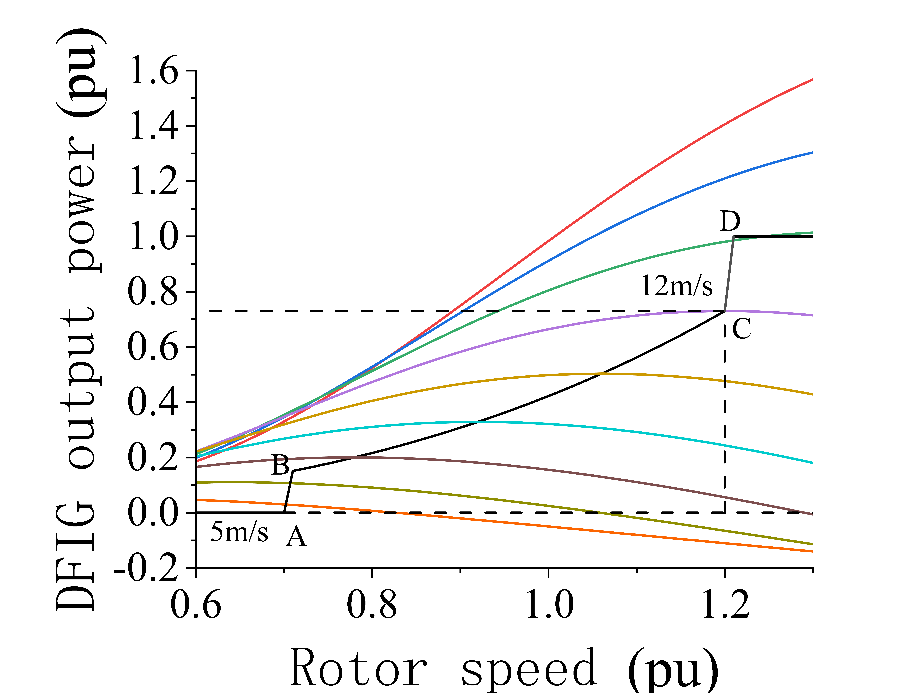


S3 Fig. Control block diagram of the rotor-side controller based on stator voltage orientation


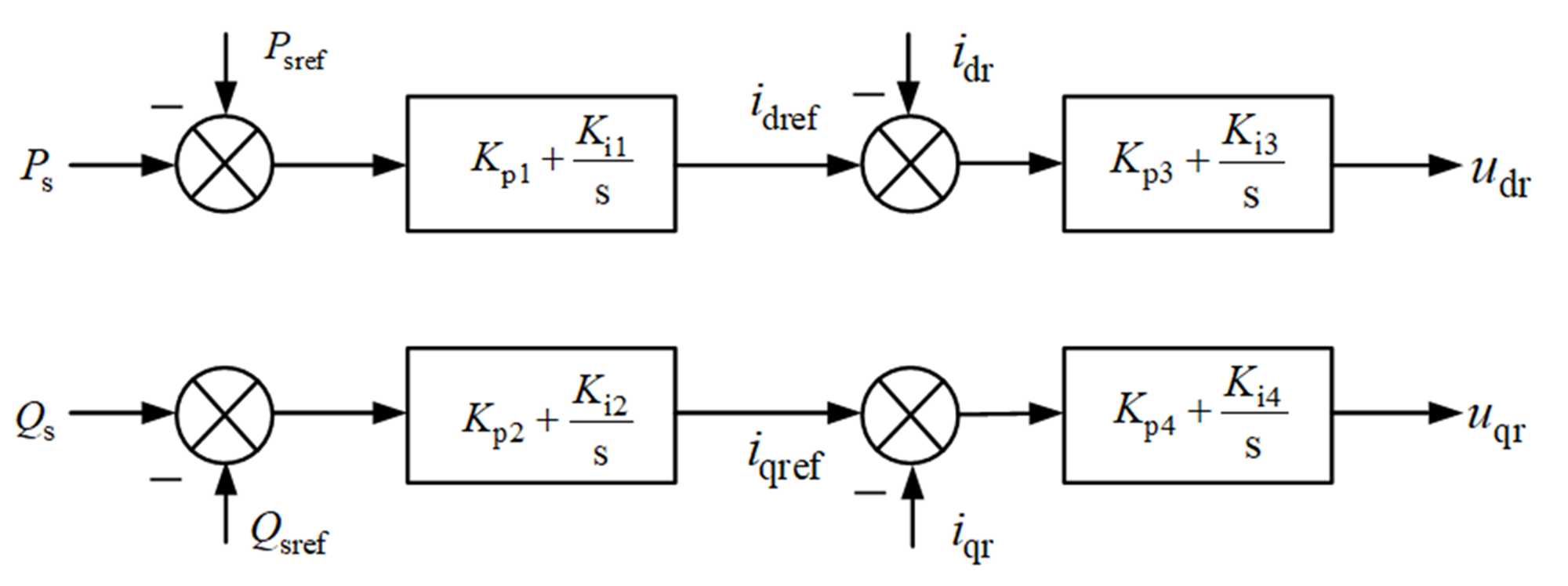


S4 Fig. The process of optimizing DFIG PID parameters using improved NSGA-II


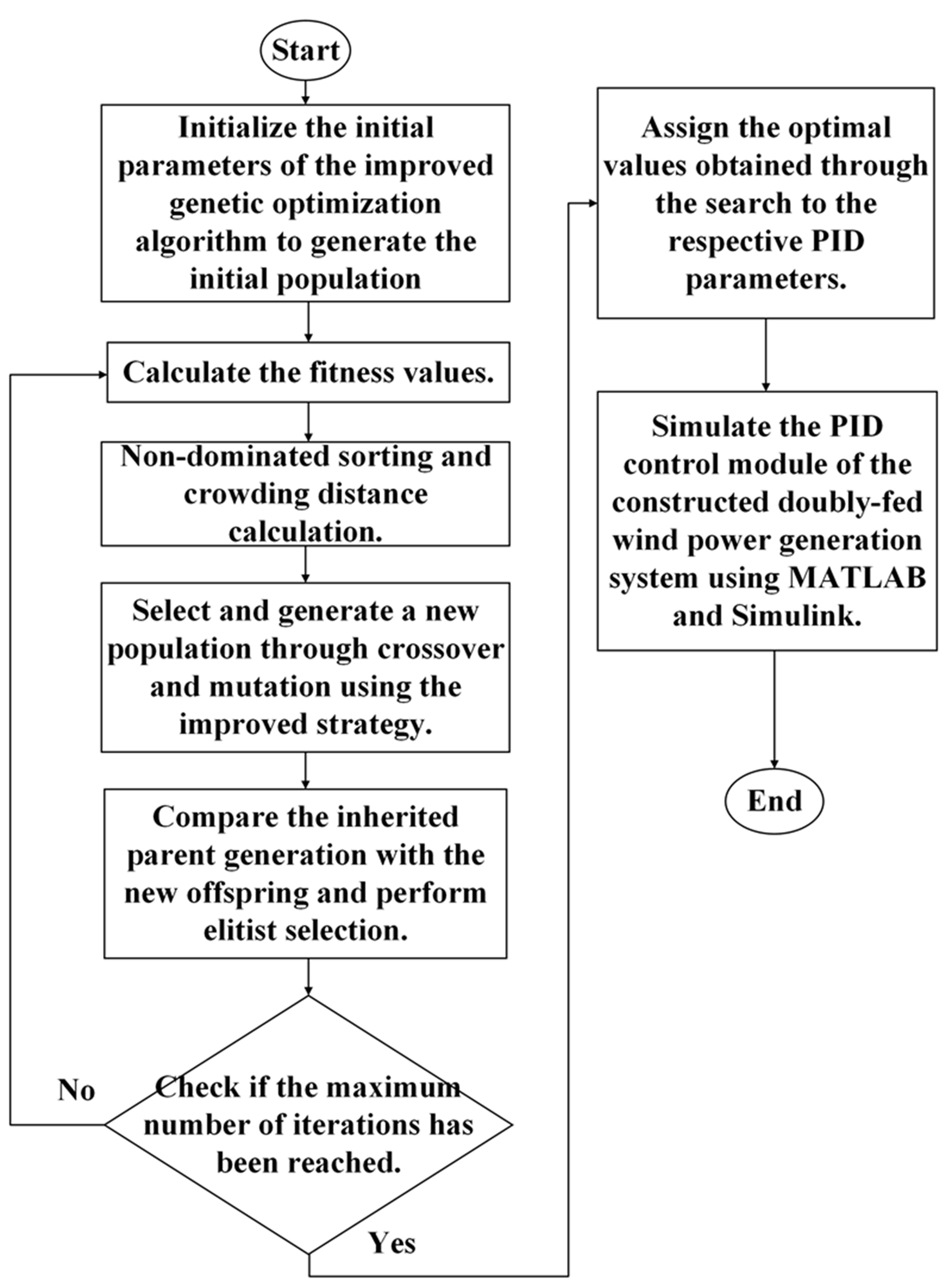


Fig 5: DC bus voltage variations


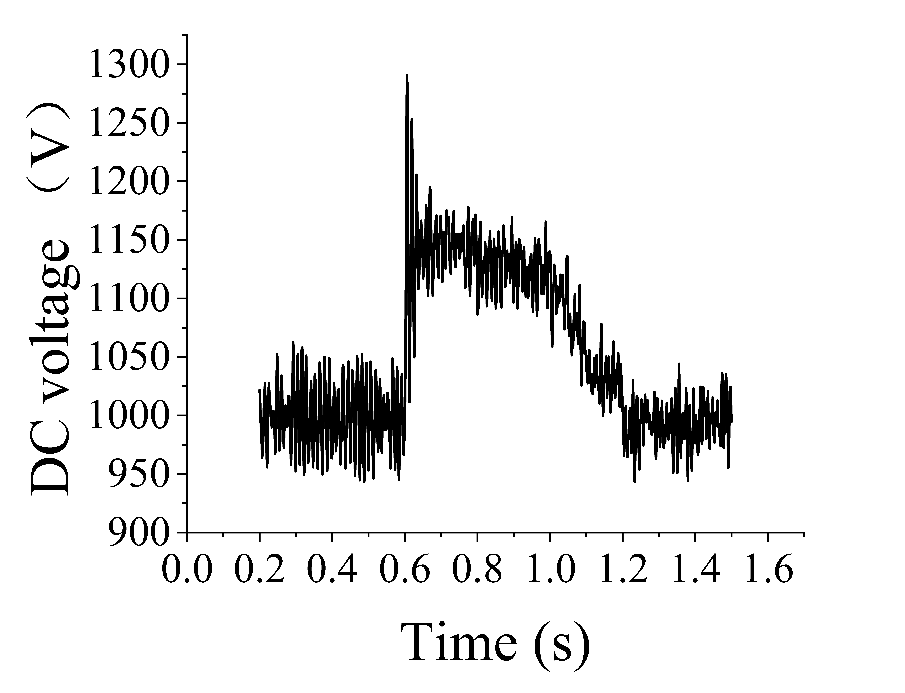


S6 Fig. Rotor-side three-phase current


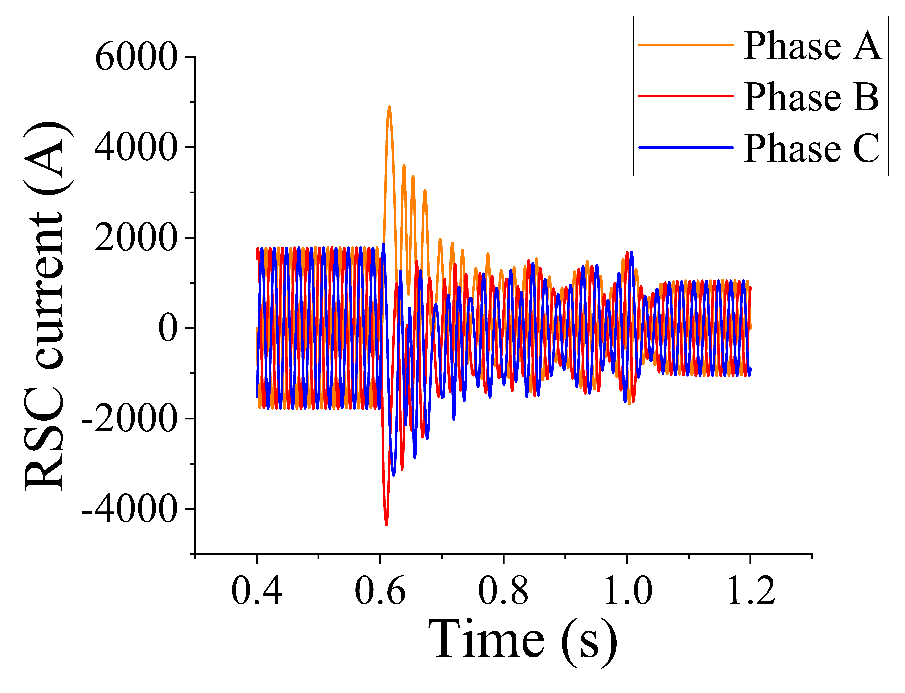


S7 Fig. Effect of different Kp values on the rotor-side current with Ki = 1


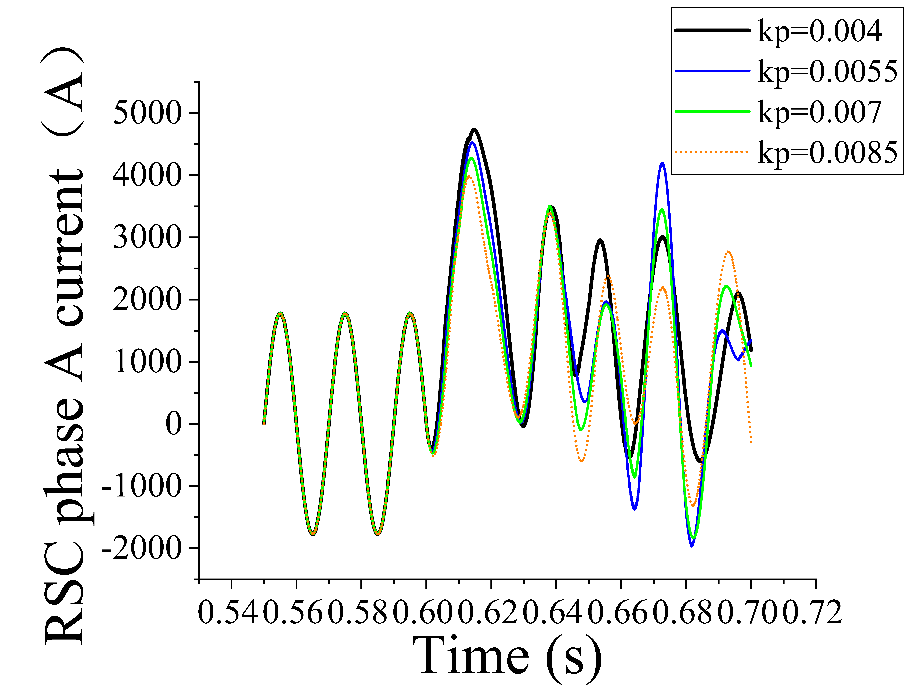


S8 Fig. Effect of different Ki values on the rotor-side current with Kp = 0.008


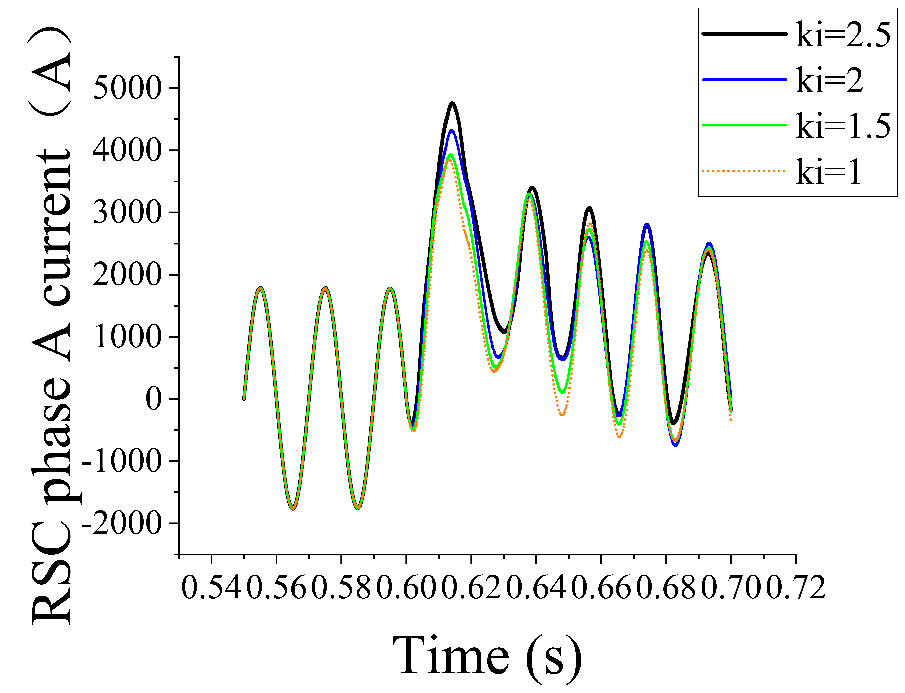


S9 Fig. Fitness value convergence of the four optimization algorithms


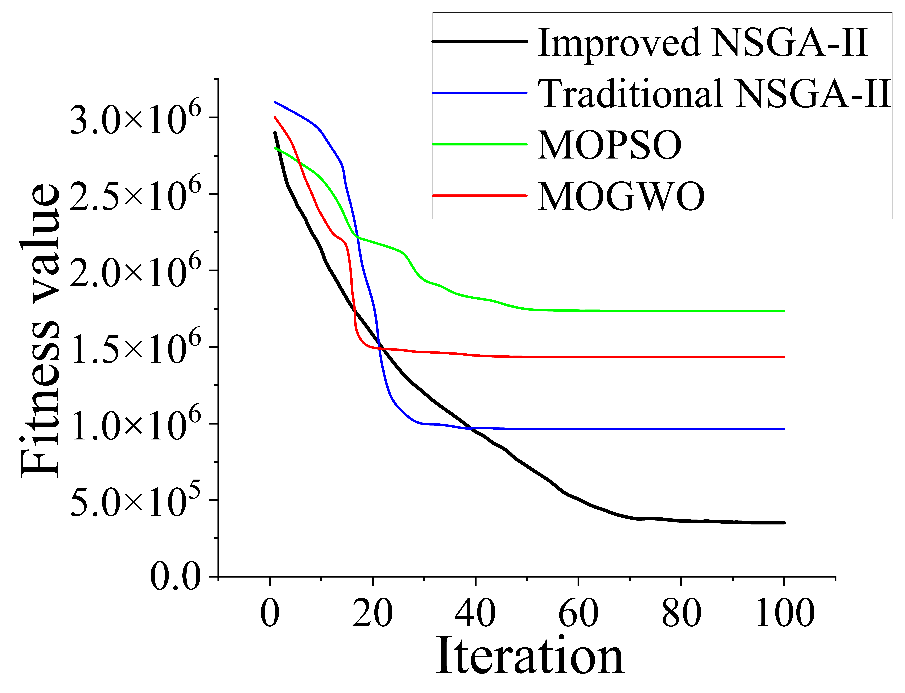


S10 Fig. Comparison of the rotor-side phase-A current at 1.2 times the nominal voltage after optimization using the different algorithms


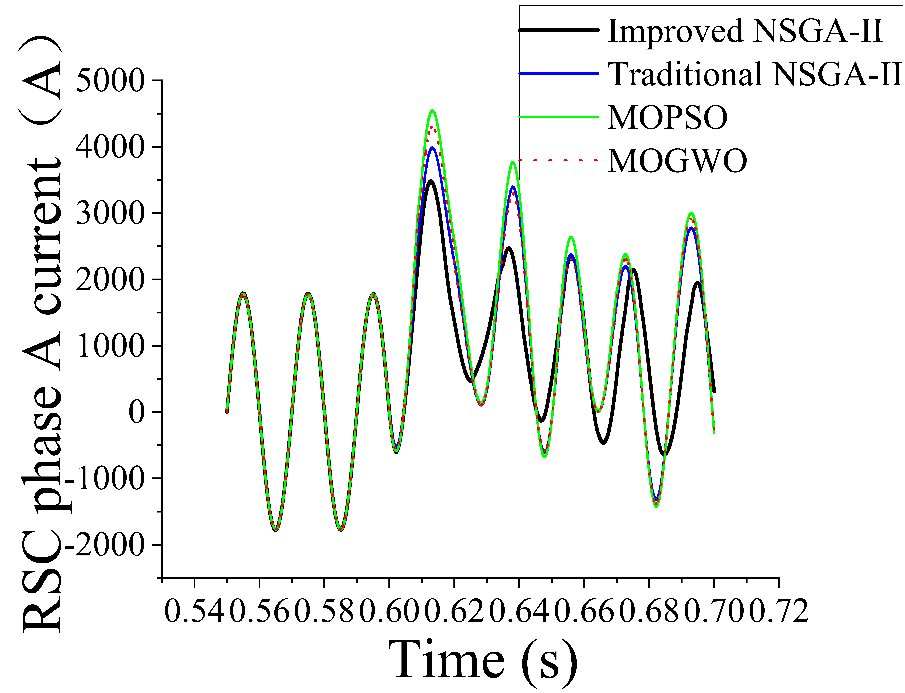


S11 Fig. Current comparison at 1.3 times the nominal voltage boost after parameter optimization using the different algorithms


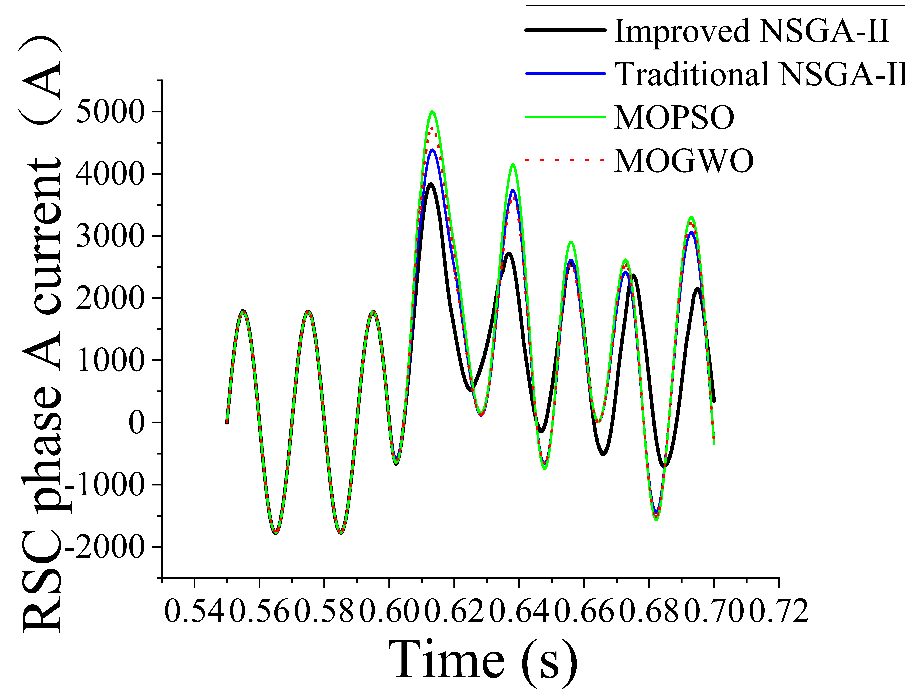


S1 Table. Parameter values

| Parameter | Value |
| --- | --- |
| K_pmin_ | 0.005 |
| K_pmax_ | 2 |
| K_imin_ | 0.5 |
| K_imax_ | 5 |

S2 Table. Key parameters of DFIG

| Parameter | Value |
| --- | --- |
| Rated capacity | 1.5 MW |
| Stator reactance | 0.08 pu |
| Rated voltage | 0.69 kV |
| Rotor reactance | 0.09 pu |
| Rated frequency | 50 Hz |
| Mutual reactance | 2.75 pu |
| Stator resistance | 0.69 kV |
| DC capacitor | 0.09 pu |
| Rotor resistance | 50 Hz |
| DC bus-rated voltage | 2.75 pu |

S3 Table. Optimized PID controller parameters obtained using the four optimization algorithms

| Parameter | Traditional NSGA-II | Improved NSGA-II | | MOPSO | MOGWO |
| --- | --- | --- | --- | --- | --- |
| *K*_p_ | 0.00932 | | 0.00793 | 0.01304 | 0.01009 |
| *K*_i_ | 1.54621 | | 1.01171 | 1.92534 | 1.78623 |

S4 Table. Performance comparison of the optimization algorithms under voltage boost to 1.2 times the nominal value

| Grid voltage boosted to 1.2 times the nominal value | | Traditional NSGA-II | Improved NSGA-II | MOPSO | | MOGWO |
| --- | --- | --- | --- | --- | --- | --- |
| Current after voltage boost (first cycle) | 3965.60702 | | 3484.79077 | 4520.79200 | 4282.85558 | |
| Current after voltage boost (second cycle) | 3295.61013 | | 2473.26821 | 3771.2652 | 3397.53622 | |
| Iterations required for identifying the optimal solution | 35 | | 79 | 45 | 21 | |
| Fitness value of the optimal solution | 964019.4 | | 352215.6 | 1735103.0 | 1433592.0 | |

S5 Table. Performance comparison of the optimization algorithms under voltage boost to 1.3 times the nominal value

| Grid voltage boosted to 1.3 times the nominal value | | Traditional NSGA-II | Improved NSGA-II | MOPSO | | MOGWO |
| --- | --- | --- | --- | --- | --- | --- |
| Current after voltage boost (first cycle) | 4362.16772 | | 3833.26984 | 4972.8712 | 4711.14113 | |
| Current after voltage boost (second cycle) | 3625.17114 | | 2720.59503 | 4148.3917 | 3737.28984 | |
